# Assessment of total aflatoxin and ochratoxin A in poultry feed ingredients by thin-layer chromatography and enzyme-linked immunosorbent assay

**DOI:** 10.5455/javar.2024.k754

**Published:** 2024-03-31

**Authors:** Mustafa Rahim, Nadeem Rashid, Khanoranga, Waqas Ahmad, Zainia Rehmat, Afroz Rais, Zainab Siddique, Kashif Kamran

**Affiliations:** 1 Center for Advanced Studies Vaccinology and Biotechnology, University of Balochistan Quetta, Quetta, Pakistan; 2 Department of Environmental Science, Sardar Bahadur Khan Women’s University, Quetta, Quetta, Pakistan; 3 University of Veterinary and Animal Sciences, Lahore, Narowal Campus, Pakistan; 4 Department of Microbiology, Sardar Bahadur Khan Women’s University, Quetta, Pakistan; 5 Department of Botany, Sardar Bahadur Khan Women’s University, Quetta, Pakistan; 6 Department of Zoology, Sardar Bahadur Khan Women’s University, Quetta, Pakistan; 7 Department of Zoology, University of Balochistan, Quetta, Pakistan

**Keywords:** Thin layer chromatography, viable fungal count, total aflatoxins, ochratoxin A

## Abstract

**Objective:**

This study was conducted to evaluate the prevalence of total aflatoxin (AF) and ochratoxin A (OTA) in poultry feed ingredients under different environmental conditions during the summer and winter seasons, while the hygiene quality of the feed ingredient was assessed through viable fungal count (VFC).

**Materials and Methods:**

A total of 288 poultry feed ingredients (*n =* 96 each) samples were collected from different poultry shops, which were initially analyzed for the presence of AF and OTA through thin layer chromatography (TLC) and then confirmed the contamination concentration through the enzyme-linked immunosorbent assay method.

**Results:**

The results of the current study confirmed the incidence of contamination with AF and OTA by TLC and ELISA methods. The contamination level of AF ranged from 26.09 to 50.56 (mean* =* 41.22 ± 9.45) μg/kg, whereas the contamination level of OTA ranged from 50.13 to 6.21 (mean 42.60 ± 6.21) μg*/kg.* The contamination level of AF was found to be above the permissible level set by the Food and Drug Administration (20 μg/kg), whereas the contamination level of OTA was below the permissible limits. Moreover, the VFC values were also below the recommended level. The results showed that the association between AF, OTA, and moisture content was significant (*p* < 0.05).

**Conclusion:**

Mycotoxin contamination was significantly (*p* < 0.05) highest in the winter season. These findings suggested that continuous monitoring regimes might prevent mycotoxin contamination in poultry feed ingredients.

## Introduction

Mycotoxins are secondary metabolites of fungi that are commonly found in agricultural products. Total aflatoxins (AF) and ochratoxins A (OTA) are the most widespread mycotoxins and are of great concern in humid climates such as Pakistan and India [1]. These mycotoxins are released by fungi like *Aspergillus* and *Penicillium* species that usually contaminate poultry feed ingredients [2]. Aflatoxins are the most focused toxins due to their link with a high rate of mortality and morbidity in animals. The presence of aflatoxins in poultry feed ingredients is considered a main problem for human health, adversely affecting the health of animals and humans. Due to the great risk of mycotoxin contamination in feed ingredients, it is regarded as an unavoidable contaminant and has been designed to have regulatory limits for poultry (20 μg/kg) [3]. Mycotoxins are posing a serious threat to livestock and consumers due to their toxicity and synergistic characteristics [4]. Animals ingesting contaminated feeds with a high concentration of mycotoxins showed various effects on their bodies, such as reduced production and suppression of the immune system [5]. Many outbreaks of mycotoxicosis diseases have been reported in humans and animals due to the consumption of mycotoxin-contaminated food and feed [6]. Pakistan is characterized by a diverse climate in different areas. The favorable climate is likely to increase the chance of mycotoxin production in different animal feeds (especially poultry feed). A few studies in Pakistan have also reported the presence of mycotoxins in poultry [7]. In Pakistan, the poultry industry is dependent on the quality of feed produced locally. It is a common practice that feed mill owners buy a huge bulk of grains during production seasons, and that bulk of grain is used throughout the year. Long post-harvest periods and improper storage conditions in a warm and moist environment increase the chance of fungal invasion in feeds, eventually leading to mycotoxins production [8].

Monitoring of mycotoxins in feed ingredients is vital to assess the degree of exposure in chickens and may help reduce the risk of exposure in people. This is because contamination without regulatory measures can have dangerous effects. Therefore, the specific objectives of the present study were (i) to evaluate the degree of contamination of AF and OTA widely used in feedstuffs (corn, rice, and wheat) and (ii) to study the climatic effects on the occurrence of the studied mycotoxins. (iii) Viable fungal count (VFC) was also carried out to check the hygienic quality of poultry feed ingredients.

## Materials and Methods

### Ethical approval

Ethical approval (No. 59/CASVAB) was taken from the ethics committee of the Centre for Advanced Studies in Vaccinology and Biotechnology, University of Balochistan, Quetta.

### Sample collection

Samples of broiler feed ingredients (corn, broken rice, and wheat) were randomly collected from different animal feed shops in Quetta from September 2021 to August 2022. Sampling was done following the method of Richard [9], with little modification. 500 gm of sample from a lot was collected by several subsamples (100 gm) and mixed to form a uniform sample. The feed ingredients (total 288, *n =* 96 each) were collected in sterilized polythene bags and were immediately brought to the Toxicology Laboratory of the Center for Advanced Studies in Vaccinology and Biotechnology, University of Baluchistan, Pakistan, for further examination. Then these samples were ground, mixed, and stored at 2°C–8°C for extraction.

### Sample extraction and cleanup method

Extraction was carried out with 70% methanol. Five grams of ground sample were mixed with 25 ml of methanol and vigorously shaken for 4 min. The suspension was then filtered with Whatman filter paper (42) and stored in the cleanup step. 5 ml of the filtrates were passed through the Mycosep^®^ column (226), and the residues were evaporated [10].

### Thin layer chromatography (TLC)

For TLC analysis, residues were dissolved in toluene. The samples were spotted against the standard solution of AF and OTA on a TLC plate (60°C) through an autospotter. The plate of TLC was developed with chloroform and acetone (9:1). The plate was dried about 1 cm from the top, and the rest was dipped in methanol and sulfuric acid (90:10). The plate was heated at 150°C after a heating spot was seen at 365 nm UV light and then compared to standard spots of standard solutions [11].

### Enzyme-linked immunosorbent assay

Using the Direct Assay ELISA Test Kit for AF and Ochratoxin A (OTA). The enzyme-conjugated was mixed and then added to the antibody-coated. The samples and standards were allowed to compete separately with enzyme-conjugated AF and OTA. After a step of five washes, an enzyme substrate was added and blue was developed. The next step involved adding a stop solution. Absorbance was read at 460 nm by a computerized microplate reader, and AF and OTA were expressed in μg/kg [12].

### Moisture content (%)

Five grams of the feed ingredient samples (replicated three times) were dried in an oven at 100°C for 24 h. The sample was weighed, and the initial water content was determined with the help of the following formula [13]:


$$\%\;\mathrm{Moisture}=\frac{\mathrm{Loss}\;\mathrm{in}\;\mathrm{Moisture}\;(\mathrm{gm})}{\mathrm{Initial}\;\mathrm{weight}\;\mathrm{of}\;\mathrm{sample}\;(\mathrm{gm})}\times 100\mathrm{\%.}$$

### Determination of pH

Fifty grams of each sample were homogenized in 100 ml of de-ionized water for 5 min by a tissue homogenizer (Edmund Buhler 7,400 Tubigen H04), and pH was measured using a calibrated pH meter (JENWAY 3,510). The probe of the pH meter was inserted in the slurry and allowed to stay until the constant reading [14].

### VFC

A VFC was carried out using the surface spread method with few modifications [15]. Briefly, a 10-gm portion of each feedstuff sample was added to 90 ml of a 0.1% mycological peptone water solution for 30 min shaken using a reciprocal shaker (Labnet, S 2030-RC-220). Prepare tenfold serial dilution (10–2 to 10–4) from each mixture in sterile de-ionized water. A volume of 100 µl from these dilutions was spread in triplicate on the surface of the agar of Sabouraud plates and incubated at 28°C for 7 days in a dark place. Plates containing 10–100 colony forming unit (CFU). VFC was calculated, and their results were presented in CFU per gram of sample.

### Chemicals and reagents

All the used solvents were of analytical grade and were purchased from reputed companies. Samples and standards were prepared using double-distilled water. The analytical standards for AF and OTA were bought from Sigma-Aldrich. Before use, the laboratory glassware was repeatedly cleaned with ultrapure water after being stored overnight in 10% (v/v) nitric acid (Merck, Germany).

### Quality control and quality assurance

Following stringent quality assurance and control procedures, methodological validity was attained. The real samples and procedural blanks were all examined in the same manner. Blank samples were used to assess cross-contamination and interference in real samples. After each set of feed ingredient samples, blanks were analyzed concurrently, and the ELISA kit was calibrated before each batch of samples. A recovery study was used to validate the analytical approach by spiking samples with known concentrations of AF and OTA standards. The average recoveries (%) for AF and OTA were 92.30 ± 6.17 and 91.63 ± 5.43, respectively. The limit of detection for AF and OTA was 1.05 and 1.79 µg/kg, whereas the limit of quantification for AF and OTA was 3.81 and 5.28 µg/kg, respectively.

### Data analysis

Samples were analyzed in triplicate, and means were then calculated for further data interpretation with a standard deviation. The mean of variance was carried out for investigating parameters through a one-way analysis of variance (ANOVA) by using SPSS (20.00), and basic statistics were carried out in Excel (2010).

## Results

**Figure 1.**
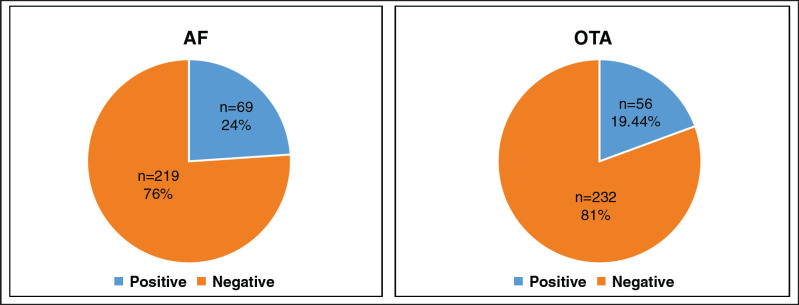
Frequency of AF and OTA in analyzed samples of the study area.

**Figure 2.**
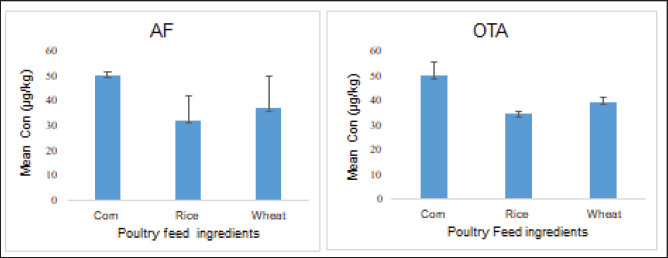
Level of mean concentration of the studied mycotoxins in the poultry feed ingredients of the study area.

The results of the current study revealed that contamination of AF has been found positive in 69 out of 288 total feed ingredient samples. While the OTA has occurred in 56 samples (19.44%) of poultry feedstuffs (Fig. 1), the highest mean concentration (μg/kg) of AF contamination was reported in corn (50.29 ± 31.34), while the lowest concentration (μg/kg) was found in rice (32.41 ± 19.61) (Fig. 2). The highest mean concentration (μg/kg) of OTA contamination level was detected in corn (50 ± 5.45), whereas the lowest level was observed in rice (34.56 ± 1.23) (Fig. 2).

### Assessment of physical properties and VFC

The samples of feed ingredients were analyzed. Physical properties (moisture, pH) and VFC values are presented in Table 1. It is observed that the moisture content (%) in poultry feedstuffs ranged from 12.37 ± 1.13 to 15.21 ± 0.76. Overall, moisture content was recorded as higher in the winter season (Table 1). The pH of the analyzed samples for both seasons (winter and summer) ranged from 5.53 ± 0.37 to 5.59 ± 0.34. The mean pH was recorded at 5.61 in the winter season and 5.48 in the summer season. The VFC was observed to be significantly (*p* < 0.05) higher in corn (1.45 × 10^3^ ± 2.64) during the summer season. Meteorological data (Table 2) of the study area revealed that the mean temperature (°C) of the season was highest at 40.36°C ± 1.2°C recorded in summer and in winter at 16.55°C ± 0.63°C, whereas humidity at 40.33% ± 0.34% in summer and in winter was 58.33% ± 2.54% recorded, and rainfall measured in summer and winter was 12.33 ± 2.92 and 51 ± 1.12 mm, respectively.

### Correlation between pH and mycotoxins

In the current study, Pearson’s correlation coefficient analysis was carried out to assess the impact of pH on the level of analyzed mycotoxins in poultry feed ingredients. The results showed that the *R*^2^ value for AF was 0.0406, whereas for OTA the *R*^2^ value is 0.067, which indicates no relationship between pH and AF or OTA.

### Seasonal variations in the studied parameter

**Figure 3.**
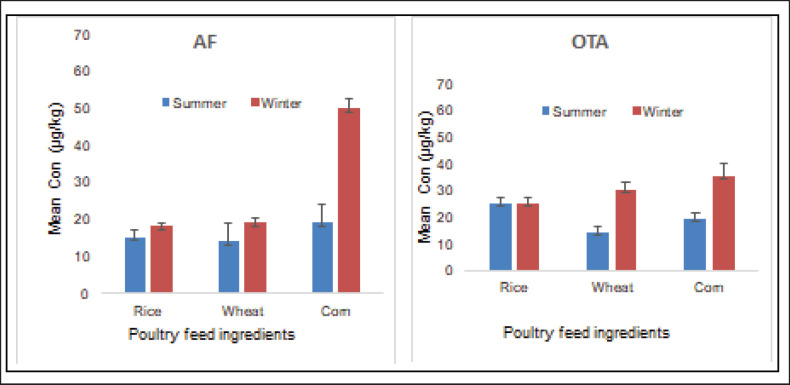
Seasonal variations in investigated mycotoxins in poultry feed ingredients.

The prevalence of mycotoxins in different poultry feed ingredients is investigated in different seasons (summer and winter). The result of the study showed a higher concentration of AF and OTA in the winter season (Fig. 3). Differences in the level of the studied mycotoxins between summer and winter were statistically significant (*p* < 0.05) (Table 3). Similarly, moisture content and VFC also showed significant seasonal variations, whereas non-significant (*p* > 0.05) seasonal variations were observed in the pH of the analyzed samples.

**Table 1.** Moisture content (%), pH and VFC of different feed ingredients at the study area.

Season	Feed ingredients	Moisture mean ± SD	pH mean ± SD	VFC mean ± SD
Summer	Corn	15.05 ± 0.63	5.51 ± 0.38	1.45 × 10^3^ ± 2.64
	Rice broken	12.65 ± 1.03	5.48 ± 0.33	9.33 × 10^2^ ± 0.13.6
	Wheat	12.61 ± 1.2	5.53 ± 0.30	1.17 × 10^3^ **±** 2.8
Winter	Corn	15.51 ± 0.26	5.61 ± 0.39	1.51 × 10^3^ **±** 2.54
	Rice broken	13.08 ± 0.76	5.61 ± 0.39	1.03 × 10^2^ **±** 1.63
	Wheat	12.33 ± 1.10	5.53 ± 0.30	1.03 × 10^3^ ± 2.1

**Table 2.** Meteorological data of sampling areas during study time frame (2021–2022).

Seasons	Temperature (°C)	Humidity (%)	Rainfall (mm)
Summer Range	42–40	41–42	12–14
Mean ± S.D	40.36 ± 1.2	40.33 ± 0.34	12.33 ± 2.92
Winter Range	14–26	50–65	49–56
Mean ± S.D	16.55 ± 0.63	58.33 ± 2.54	51 ± 1.12

## Discussion

Pakistan is an agricultural country with a diverse climate, and the majority of the area (almost two-thirds) falls into the arid type [16]. Wheat, corn, and rice, with productions of 27,634, 10,183, and 7,322 tons, respectively, were the major cereal crops during the year 2022 [17]. Occasionally, the yield of these crops exceeds the need for human consumption at the national level and is not only exported but also used as a feedstuff for animal feeding [18]. Moisture contents, total fungal count, and mycotoxin contamination are important indicators of the hygienic quality of agro-feedstuffs intended for animal consumption. The fungus’s growth mainly depends on the moisture content and pH of the substrate [19]. The current study indicated a significant (*p* < 0.05) increase (*p* < 0.05) in moisture contents, pH, and fungal and mycotoxin contamination in the studied feedstuff. The overall data of the analyzed feed stuff, including corn, rice, and wheat, revealed moisture contents (Mean ± SD) of 15.2 ± 0.8, 12.8 ± 0.9, and 12.4 ± 1.1, respectively [18]. evaluated feed stuff, viz*.,* corn, wheat, and rice, and reported moisture contents ranged from 6.72% to 15%. The moisture contents in corn were recorded at 10.5% to 15%, in wheat they ranged from 6.72% to 8.32%, and in rice they ranged from 9.19% to 11.10%, respectively. Moisture contents of a feedstuff are good markers of the quality and shelf life of grains. The moisture contents of grains higher than 14% support fungal growth, and the mycological contamination higher than 13% not only reduces the grain quality but may also result in mycotoxin production [20]. Current data revealed that the overall pH of the sampled feedstuff was slightly acidic (6.45–6.68). Stuff-wise results indicated the pH range of feedstuff “corn” was found to be 6.51–6.68. Similar findings were reported [21], who analyzed corn and reported a pH range of 6.0–6.59. Findings of the present study concerning feedstuffs “rice broken” and “wheat revealed a pH range of 6.48–6.61 and 6.45–6.53, respectively [22]. Determined the pH and reported a range between 6.48 and 6.61 for broken rice and 6.45 and 6.53 for wheat, and the observations of the above-mentioned researchers are quite consistent with the present study. Mycological growth greatly depends upon the pH of culture media, and a slightly acidic pH supports fungal growth [21]. The pH value of the growth medium affects fungal growth either by direct action on cells or indirectly altering nutrient availability [23].

**Table 3.** One-Way ANOVA (*p* < 0.05) performed to evlaute variations in the studied parameters.

Variable	Seasons	Feed type
Total Aflatoxin	0.000*	0.000*
Ochratoxin A	0.000*	0.001*
Moisture Content	0.003*	0.001*
pH	0.06	0.07
Viable fungal Count	0.000*	0.001*

*Values are shown as statistically significant at *p* < 0.05.

The current study conveys the findings of investigations regarding the mycological contamination of different poultry feedstuffs. The data about the mycological contamination concerning feedstuff “corn and rice broke” showed fungal contamination significantly (*p* < 0.05) higher in the winter season. The current findings agree with other studies that observed a higher fungal count in the samples of poultry feedstuff collected during the winter season [24,25]. Mycological analysis of the present study further indicated significantly (*p* < 0.05) higher fungal counts in the summer season collected feedstuff “wheat.” Similarly, significantly (*p* < 0.05) higher VFC were recorded in the wheat samples collected during the summer season [25]. A VFC is used to reveal the overall mycological contamination and the level of hygiene of feedstuffs [23]. In the current study, VFCs were recorded within the permissible limits (1 × 10^4^ CFU gm^−1^) set by manufacturing practice. Similar findings were also reported with VFC below the allowable limit [21]. On the contrary, a study conducted in Argentina reported VFC above the permissible limit and mycological contamination of agricultural products may occur during pre-harvest and post-harvest stages [24]. The frequency of fungal contamination varies with geographical location, season, and abiotic factors, including moisture contents and pH [26,22].

Cereals are the mainstay for the provision of metabolizable energy in poultry feed formulations and agricultural commodities, including corn, rice, and wheat, due to their chemical composition, which may serve as a suitable culture medium for fungal growth and the subsequent release of mycotoxins [27]. The current study revealed mycotoxin (AF) contamination in the analyzed feedstuff. Overall, 42.7% of corn, 18.70% of rice, and 26% of wheat samples were recorded as mycotoxin-contaminated, and among the contaminated, 4.2% of corn samples showed contamination higher than the permissible levels of 20 pbb set by the pakistan standards and quality control authority (PSQCA). Whereas the level of AF contamination of rice and wheat was within the PSQCA permissible limits [28]. Evaluated from Jordan AF in feedstuffs, including corn, rice, and wheat, and reported contamination in the observed samples. However, all the positive samples were within the Food and Drug Administration (FDA) permissible limit of 20 μg/kg. The present study also revealed OTA contamination in the analyzed feedstuff. The data indicated that corn, rice, and wheat samples indicated OTA contamination (34%, 21%, and 21%, respectively. Furthermore, all the analyzed samples were within the permissible limits of 100 µg/kg proposed by the European Union [29]. They collected 74,821 feed samples from 100 different countries and stated that 15% of the samples were OTA-positive. Another study from Nigeria tested mycotoxin contamination in feedstuffs including maize and wheat and reported OTA at a level of 10 μg/kg [20]. Contamination in Pakistan has also been reported by many researchers in this regard [25]. OTA contamination in agriculture commodities, viz*.,* corn, corn kernels, rice, and wheat, intended for human consumption [27]. Pakistan evaluated OTA contamination in feedstuff samples intended for poultry use and reported that 40% of corn, 30% of rice, and 36% of wheat samples showed OTA contamination with the mean level of OTA concentration below the upper limit suggested in EC guidelines.

Mycotoxin contamination has been considered an unavoidable contaminant of agricultural products [27], and internationally, about 25% of agro-products are reported to be contaminated with mycotoxin. Certain environmental conditions and physical characteristics, such as humidity, storage temperature, moisture content, and pH of staff, play key roles in enhancing the growth of mycotoxigenic fungi and subsequent mycotoxin release [13]. Climate change and inadequate awareness regarding standard cultivation, handling, and storage procedures also critically affect the growth of mycotoxigenic fungi and mycotoxin production [22]. Occurrences of mycotoxins in agricultural produce countries are more likely in the hot and humid regions [20]. Pakistan, being located in Southeast Asia with mycotoxin-producing fungi and a conducive climate [30], is among the countries at high risk for agricultural mycotoxin contamination.

## Conclusion

The current study revealed the prevalence of AF and OTA in feedstuffs intended for animal use. The analyzed feedstuff samples revealed seasonal variation in the occurrence and mean concentration of mycotoxins. Overall, a higher percentage of positive samples and higher levels of mycological contamination and concentrations of mycotoxins were observed in the winter season. The mean concentration of analyzed mycotoxins was recorded within the FDA’s permissible limits, except for a few samples. The occurrence of potentially toxic mycotoxins in the feed ingredients seems to be a serious health and economic threat for consumers. The need for constant monitoring, follow-up, and adaptation of corrective actions is suggested.

## List of Abbreviations

AF, Total aflatoxin; CFU, Colony forming unit; FDA, Food and Drug Administration; OTA, ochratoxin A; PSQCA, Pakistan standards and quality control authority; TLC, *Thin layer chromatography;* VFC, Viable fungal count
